# The Effects of Serotonin in Immune Cells

**DOI:** 10.3389/fcvm.2017.00048

**Published:** 2017-07-20

**Authors:** Nadine Herr, Christoph Bode, Daniel Duerschmied

**Affiliations:** ^1^Cardiology and Angiology I, Heart Center, Faculty of Medicine, University of Freiburg, Freiburg, Germany

**Keywords:** 5-hydroxytryptamine, serotonin, serotonin receptors, immune cells, immune system

## Abstract

Serotonin [5-hydroxytryptamine (5-HT)] plays an important role in many organs as a peripheral hormone. Most of the body’s serotonin is circulating in the bloodstream, transported by blood platelets and is released upon activation. The functions of serotonin are mediated by members of the 7 known mammalian serotonin receptor subtype classes (15 known subtypes), the serotonin transporter (SERT), and by covalent binding of serotonin to different effector proteins. Almost all immune cells express at least one serotonin component. In recent years, a number of immunoregulatory functions have been ascribed to serotonin. In monocytes/macrophages, for example, serotonin modulates cytokine secretion. Serotonin can also suppress the release of tumor necrosis factor-α and interleukin-1β by activating serotonin receptors. Furthermore, neutrophil recruitment and T-cell activation can both be mediated by serotonin. These are only a few of the known immunomodulatory roles of serotonin that we will review here.

## Introduction

### Peripheral versus Central Serotonin

**Figure 1:**
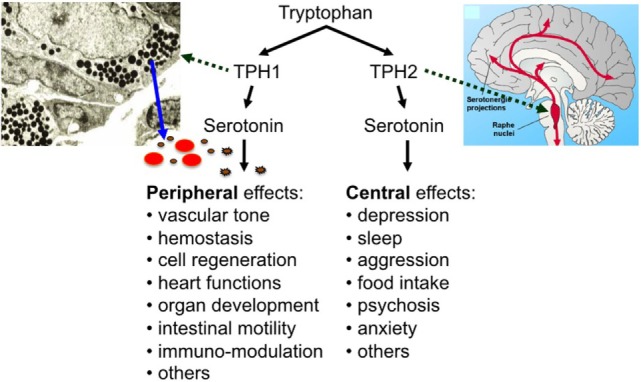
The effects of peripheral and central serotonin.

Serotonin [5-hydroxytryptamine (5-HT)] has two lifes: as a neurotransmitter, it regulates sleep, appetite, mood, and other important brain functions and—separated by the blood–brain barrier and synthesized in a different way—it plays a central role in many other organ systems as a peripheral hormone (Figure 1) (1, 2). In fact, most of the body’s serotonin is circulating in the bloodstream, transported by blood platelets (3). Most of the peripheral serotonin is synthesized by TPH1 in the enterochromaffin cells of the intestine, secreted into the bloodstream, and then taken up by circulating platelets (4). Platelets store serotonin at very high concentrations in their dense granules (at 65 mM) and secrete it upon activation (5). Resting plasma serotonin concentrations (around 10 nM) can rapidly increase to 10 µM or more when platelets become activated at the site of thrombus formation or inflammation (6, 7).

Discovered by Rapport et al. in 1948 as a vasoconstrictor (8), new functions of serotonin have since been described continuously. These functions are mediated by members of the 7 known mammalian serotonin receptor subtype classes (15 known subtypes), the serotonin transporter (SERT), and by covalent binding of serotonin to different effector proteins—named “serotonylation” by Walther, Bader, and colleagues (3, 9). Peripheral serotonin is involved in the regulation of hemostasis, heart rate, vascular tone, intestinal motility, cell growth in liver, bone, and pulmonary arteries, and the development of heart, brain, and mammary gland (3). In addition, a number of immunoregulatory functions have been ascribed to serotonin (as described below).

Theoretically, either peripheral—i.e., predominantly platelet-derived—or central—i.e., neuronal—serotonin (or both) could modulate immune responses. In their review article, from 1998, Mössner and Lesch discussed the possibility of a neural-immune interaction *via* the autonomic nervous system, but found only two of four criteria to be fulfilled in the case of serotonin (6): serotonin receptors are present on immune cells and serotonin has immunoregulatory effects. Two other criteria do not apply in the case of serotonin, though. One criterion is the local association of neurotransmitter-specific nerve fibers with immune cells (although serotonin can be taken up by noradrenergic terminals on smooth muscle cells, similar to the adrenal medulla) (10, 11). The other criterion is the exclusive neurotransmitter supply of the immune target cells/organ by neurons, i.e., that the target organ could be depleted of serotonin by denervation. It is hence more likely that serotonin derived from non-neuronal sources exerts most of the immunoregulatory effects. In accordance, Roszman et al. concluded from several studies that the immunomodulatory effects of serotonin are mediated primarily through peripheral mechanisms directed toward circulating immune cells (2). Possible sources for peripheral serotonin are plasma (at rather stable, nanomolar levels), monocytes/macrophages, lymphocytes, vascular smooth muscle cells, adipocytes, mast cells (although human mast cells were long thought not to contain serotonin), and platelets (6, 12–15). Local mast cells (probably rodent as well as human) produce, store, and release serotonin into the extravascular space—in part, even under neural control (6, 16, 17). Still, the vast majority of total peripheral serotonin is stored in platelets and released upon platelet activation (reaching micromolar levels) (3, 5). At least intravascular effects are, therefore, certainly mediated by platelet serotonin.

### Platelet Serotonin in Immune Responses

In 1960, Davis et al. observed that serotonin, platelets, and inflammation were closely linked: within the first minute after injection of a lethal dose of *E. coli* endotoxin, they observed a sharp decrease in platelet count and serum serotonin, accompanied by a transient increase in plasma serotonin in dogs (18). It is now known that platelets (as transport vehicles) ensure the targeted release of serotonin in platelet-activating environments like a thrombus or an inflammatory reaction. At inflammatory sites, not only soluble factors like platelet-activating factor, complement anaphylatoxin C5a, and IgE-containing immune complexes but also bacteria or parasites as well as platelet–endothelial interactions activate platelets, resulting in serotonin secretion (6, 19–22). Serotonin was shown to exert functions in innate as well as adaptive immunity. Serotonin stimulates monocytes (23) and lymphocytes (24) and hence influences the secretion of cytokines. Vascular smooth muscle cells respond to serotonin by synthesizing interleukin (IL)-6, a possibly atherogenic mechanism (25). In contrast to these descriptions of a pro-inflammatory function of serotonin, specific activation of the 5-HT2A receptor subtype in primary aortic smooth muscle cells presents a superpotent inhibition of tumor necrosis factor (TNF)-α-mediated inflammation (26). This effect was also shown *in vivo* in an animal model. The systemic selective activation of the 5-HT2A receptor with (R)-DOI blocks the systemic inflammatory response by downregulating the expression of pro-inflammatory genes and preventing the TNF-α-induced increase of circulating IL-6 (27).

Several other seemingly contradictory findings underline the complexity of peripheral serotonin effects. Two conflicting reports describe the interaction between leukocytes and inflamed endothelium upon serotonergic intervention. Kubes and Gaboury showed in 1996 that perivascular mast cells, which are believed to rapidly internalize serotonin and also to synthesize serotonin *via* TPH1, secrete serotonin to induce an early, leukocyte-independent phase of edema formation (16, 28, 29). The recruitment of leukocytes did not seem to depend on (mast cell-derived) serotonin. In 2007, Walther et al. found that leukocyte adhesion to inflamed endothelium after injection of endotoxin depended on the activation of serotonin receptors as shown by pharmacological blockade (30). Müller et al. found in 2009 that dendritic cell migration and cytokine release was modulated by serotonin (31). In our recent studies, leukocyte recruitment to sites of inflammation is impaired in the absence of (platelet-derived) serotonin and enhanced if plasma serotonin levels rise (32, 33).

In 1999, Gershon commented the complexity of peripheral serotonin effects in an ironic way (34): “5-HT has delighted every pharmacologist who ever applied it to a gastrointestinal preparation; something always happens, no matter what the experimental circumstances. For example, depending on the conditions, 5-HT can make the bowel contract or relax, secrete, or not secrete. The problem that has bedeviled attempts to determine what 5-HT actually does for the gut has been that it is able to do too much.” In 2009, Berger even counted a “Myriad effects of serotonin outside the central nervous system” (3). The same complexity seems to apply also to the role of serotonin in immunity [Figure 3 (40)]. In conclusion, to date, the knowledge in this field remains incomplete but assigns a variety of important immunomodulatory functions to peripheral serotonin.

## Serotonergic Components of Immune Cells

Immune cells express serotonin receptors of the 5-HT1, 5-HT2, 5-HT3, 5-HT4, and 5-HT7 classes, the serotonin transporter (SERT), and the key enzymes for serotonin synthesis (TPH) and for serotonin degradation [monoamine oxidase (MAO)]. Table 1 enlists the currently known serotonergic components of immune cells.

**Table 1:** Serotonergic components of immune cells.

Cell type	5-hydroxytryptamine receptors	SERT	Tryptophan hydroxylase 1	MAO
Monocytes and macrophages	1A, 1E, 2A, 3A, 4, 7	+	+	+
Microglia	2B, 5A, 7			
Dendritic cells	1B, 1E, 2A, 2B, 4, 7	+		+
Neutrophils	(1A, 1B, 2)			
Basophils				
Mast cells	1A	+	+	
Eosinophils	1A, 1B, 1E, 2A, 2B, 6			
B cells	1A, 2A, 3, 7	+		
T cells	1A, 1B, 2A, 2C, 3A, 7	+	+	+
NK cells				
Platelets	2A, 3	+		+
Endothelial cells	1B/Dβ, 2A, 2B, 4	+	+	
Vascular smooth muscle cells	1D, 2A, 2B, 7			

Eliseeva and Stefanovich first demonstrated the presence of serotonergic receptors on leukocytes in 1982 (35). In 1988, it was shown that monocytes and macrophages take up serotonin by SERT (similar to platelets) and metabolize it to its 5-hydroxyindole acetic acid metabolite (12, 13). SERT is expressed by neurons, platelets, lymphocytes, mast cells, and monocytes (6, 36). TPH1 is expressed by enterochromaffin cells in the intestine, monocytes, macrophages, mast cells, T-cells, and endothelium (4, 6, 13, 16, 37). MAO is expressed by monocytes, macrophages, dendritic cells, T-cells, and platelets (6, 12, 13).

## Serotonin Effects on Immune Cells

### Platelets

**Figure 2:**
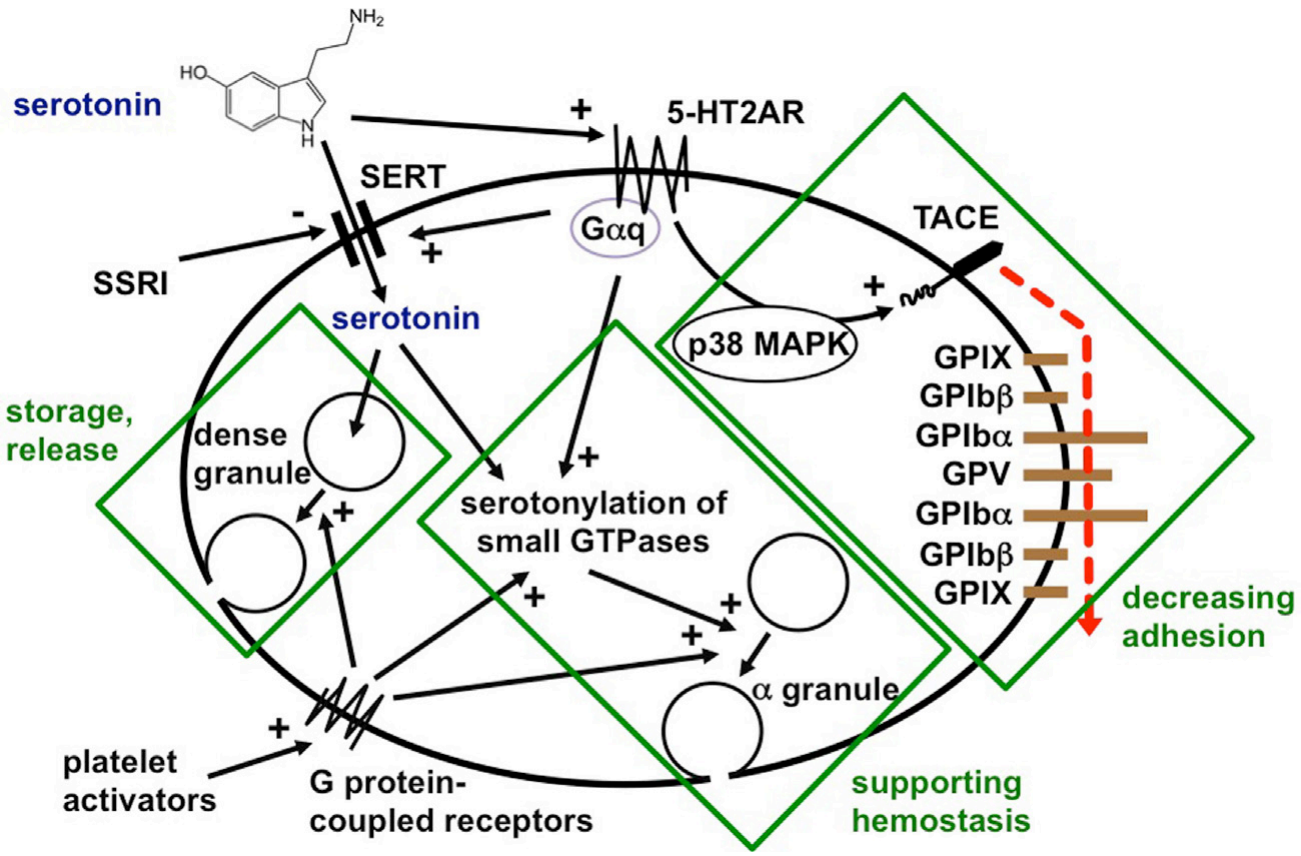
Schematic drawing of serotonin storage in dense granules, α-granule secretion, and TNF-α-converting enzyme (TACE) activation in platelets [modified from Duerschmied et al. (7)].

Platelets readily take up plasma serotonin released from intestinal enterochromaffin cells, store it, and release it after stimulation on the site of acute or chronic inflammation (Figure 2) (3, 38). In hemostasis, serotonin enhances platelet activation by weak agonists, such as ADP, after covalent binding to small GTPases by transaminases (named “serotonylation”) (9). Although 5-HT3 receptors were identified on platelets, serotonin effects on platelets have so far been ascribed to serotonylation or activation of the main platelet serotonin receptor, the 5-HT2A receptor (39). We described in 2009 that 5-HT2A receptor stimulation without further platelet stimulation induced activation of TNF-α-converting enzyme (TACE, ADAM17) (7). This resulted in shedding of the glycoproteins Ibα and V from the von Willebrand factor receptor complex and reduced platelet adhesivity. It is not known whether serotonergic platelet stimulation influences platelet-leukocyte or platelet–endothelial interactions. Platelets play a central role in delivering serotonin to inflammatory effector cells. This represents a means of highly effective targeted release of serotonin in inflamed vessels, immediately affecting circulating and resident immune cells.

**Figure 3:**
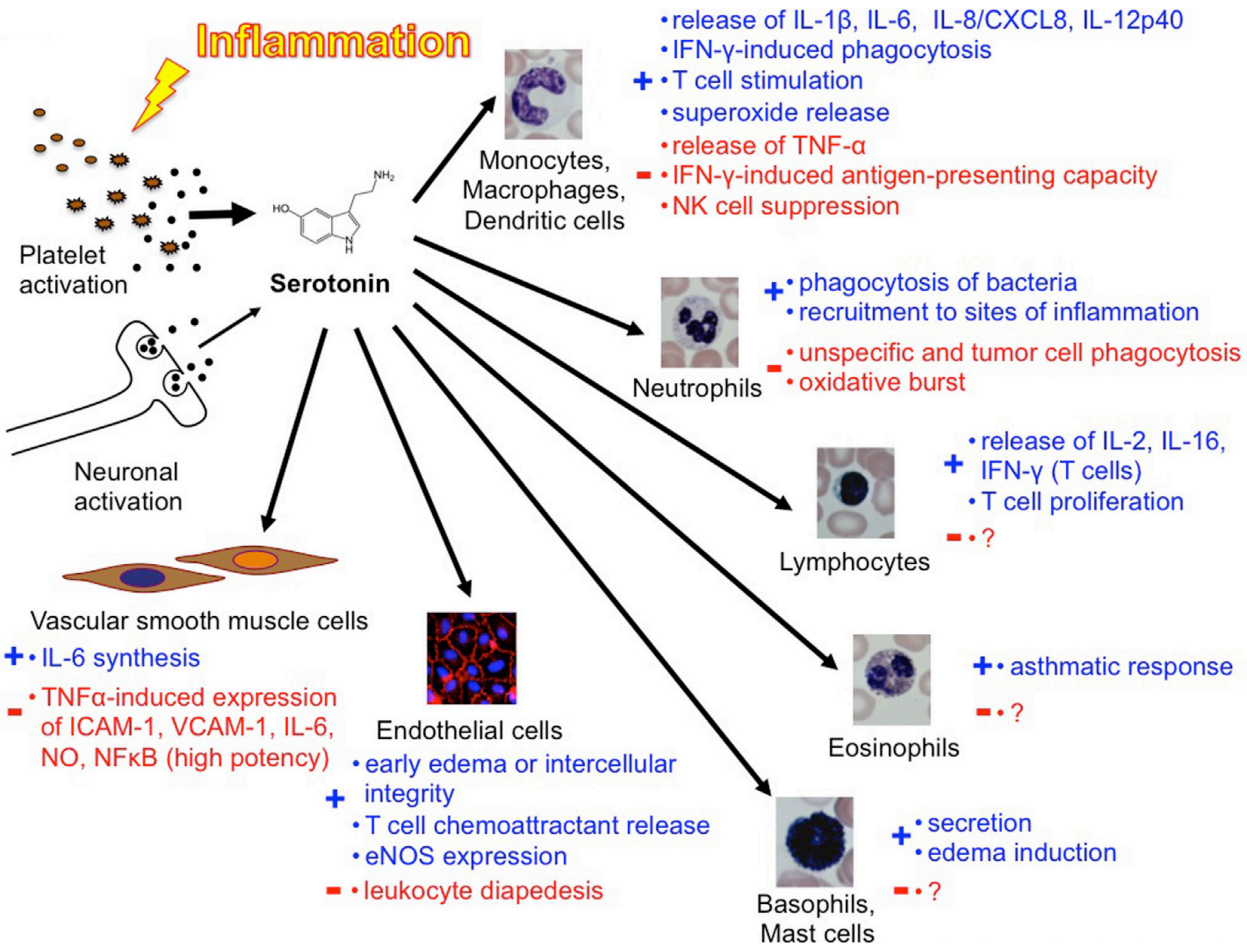
Overview of the complexity of the function of platelets and serotonin in inflammation and immunity [Duerschmied et al. (40)].

### Monocytes/Macrophages

#### Serotonergic Components

Monocytes/macrophages are believed to express the serotonergic components SERT, serotonin receptors, TPH, and MAO (Table 1) (6). Jackson et al. described in 1988 that macrophages readily take up serotonin *via* SERT (12). Sternberg et al. applied pharmacological studies in 1986 to first identify serotonin receptors of the 5-HT2 class on macrophages (41). Hellstrand and Hermodsson found in 1993 that 5-HT1A receptor stimulation on monocytes decreased the capacity of monocytes to suppress natural killer cell functions (42). We employed RT-PCR to reveal 5-HT1E, 5-HT2A, 5-HT3, 5-HT4, and 5-HT7 receptor mRNA expression in human monocytes (23). Finocchiaro et al. described serotonin synthesis as well as degradation in macrophages in 1988, suggesting that both, TPH and MAO may be expressed (13). Another pharmacological study suggested the presence of TPH, SERT, and MAO in monocytes, although the findings were controversial because blockage of serotonin synthesis with the TPH inhibitor parachlorophenylalanine (PCPA) induced an increase of serotonin in monocytes and granulocytes (43). To our knowledge, protein or mRNA expression of TPH and MAO has not been confirmed in monocytes/macrophages.

#### Serotonin Modulates Monocyte and Macrophage Function

The studies by Sternberg et al. in 1986 and 1987 showed that serotonin had inhibitory or stimulatory effects on murine macrophages, depending on the dose (41, 44). Serotonin suppressed interferon (IFN-)γ-induced phagocytosis at high, but had stimulatory effects at physiological IFN-γ concentrations. At all doses, serotonin suppressed the IFN-γ-induced antigen-presenting capacity of macrophages. Sternberg et al. also found that serotonin suppressed the IFN-γ-induced MHC class II expression but data from other groups have been conflicting (44). In 1985, Silverman et al. described specific binding of serotonin to macrophages that enhanced the release of superoxide by stimulation with phorbol myristate acetate (45).

Serotonin suppressed interactions between monocytes and NK cells, leading to an increase of NK cell functions that are normally inhibited by monocytes, such as cytotoxicity and IFN-γ production in studies by Hellstrand and colleagues in the early 1990s (42, 46, 47). When the group around Young and Young treated mice with PCPA to inhibit serotonin production in 1992 to 1995, they discovered a defect in macrophage accessory function for T cell activation (48–50): the level of expression of the alpha chain IL-2 receptor (IL-2Rα) was reduced on splenic CD4+ cells of PCPA-treated mice but not on CD8+ cells. Moreover, splenic T cell proliferation in response to concanavalin A was reduced after serotonin depletion. Both IL-2Rα expression and proliferation could be restored by exogenous serotonin and were mediated by the 5-HT2 receptor (possibly on macrophages, although this was not proven).

#### Serotonin Modulates Cytokine Secretion

Arzt et al. described in 1991 that serotonin inhibited the synthesis of TNF-α by freshly isolated and lipopolysaccaride (LPS)-stimulated human mononuclear cells (51). This effect could be inhibited by 5-HT2 receptor blockade. In 2003, Cloez-Tayarani et al. found that serotonin inhibited the production TNF-α in LPS-stimulated peripheral blood mononuclear cells and increased the release of IL-1β (52). These authors also proposed that these effects depended on stimulation of the 5-HT2A receptor. Fiebich and coworkers described in 2004 that inhibition of 5-HT3 receptors inhibited the LPS-induced release of TNF-α and IL-1β from human monocytes (53). Kubera et al. reported in 2005 that macrophages and lymphocytes needed serotonin at low concentrations for basal production of IL-6 and TNF-α, while higher concentrations of serotonin decreased the release of these markers *via* 5-HT2 receptor stimulation (54). Many of these conclusions of earlier studies that the 5-HT2A receptor was involved in several processes are based on the blockade effects of ketanserin. Ketanserin, however, is not selective for the 5-HT2A receptor and has a high affinity to block histamine H1 receptors as well and the anti-inflammatory effects attributed to the 2A receptor may in fact be due to the H1 receptor.

In contrast, involving other receptors, we found in 2005 that serotonin modulated the release of the following cytokines in LPS-stimulated human blood monocytes: IL-1β, IL-6, IL-8/CXCL8, IL-12p40, and TNF-α (23). Serotonin had no effect on the production of IL-18 and IFN-γ in our studies. The upregulation of IL-1β, IL-6, and IL-8/CXCL8 secretion was 5-HT3 receptor mediated. Activation of the 5-HT4 and 5-HT7 receptors increased the LPS-induced release of IL-1β, IL-6, IL-8/CXCL8, and IL-12p40, while, on the contrary, it inhibited LPS-induced TNF-α release. We saw no modulation of LPS-induced cytokine production at all by 5-HT1 and 5-HT2 receptor agonists.

In macrophage-like cells from human inflamed knee synovia, Seidel et al. identifed 5-HT2 and 5-HT3 receptors mediating the release of prostaglandin E_2_ but not TNF-α, IL-1β, and leukotriene B4 (55). In 2010, Tsuchida et al. reported that the prokinetic 5-HT4 receptor agonist mosapride inhibited postoperative ileus through anti-inflammatory reactions on a neuronal-macrophage axis (56). After intestinal manipulation in the rat, mosapride injection induced acetylcholine release from cholinergic myenteric neurons, which subsequently activated the α7nACh receptor on activated monocytes/macrophages. This reduced not only the further recruitment of monocytes and neutrophils but also the mRNA expression of IL-1β, IL-6, TNF-α, monocyte chemoattractant protein-1, and inducible nitric oxide synthase in the inflamed muscle layer.

In summary, the discussed studies suggest that serotonin exerts complex modulatory effects on cytokine release from monocytes and macrophages. There is especially convincing evidence that the release of TNF-α by stimulated monocytes is inhibited by serotonin. Therefore, it can be speculated that, while serotonin activates TACE in platelets, it may inhibit TACE in monocytes (7, 57). The release of IL-1β was repeatedly reported to be suppressed by serotonin.

### Microglia

Krabbe et al. found mRNA expression of 5-HT2A, 5-HT2B, 5-HT3B 5-HT5A, and 5-HT7 in brain microglia (58). They described that serotonin promoted the microglial injury-induced motility by serotonin receptor activation. In 2015, Maroteaux et al. found that the 5-HT2B receptor subtype is expressed on brain resident macrophages (microglia) and is involved in brain maturation (59). In amyotrophic lateral sclerosis, a disease associated with neuroinflammation, the upregulation of the 5-HT2B receptor was associated with slowed disease progression by less and slower degeneration of mononuclear phagocytes (60).

### Dendritic Cells

Dendritic cells express several functional 5-HT receptor subtypes that are expressed in different amounts in the different stages of maturation (61). Idzko and colleagues found in 2004 that stimulation of 5-HT3, 5-HT4, and 5-HT7 receptor subtypes mediated the release of IL-1β and IL-8 (61).

Dendritic cells are able to take up serotonin released from activated T-cells (which synthesize serotonin) and the microenvironment *via* the SERT and store it in LAMP-1+ vesicles and subsequently release it *via* Ca2+ sensitive exocytosis to promote T-cell proliferation and differentiation of naive T-cells (62). In 2009, Müller et al. described that serotonin induces oriented migration of immature dendritic cells *via* the activation of the 5-HT1 and 5-HT2 receptor subtypes. They also found that *via* the binding to 5-HT3, 5-HT4, and 5-HT7 receptors serotonin upregulates the production of IL-6 (31).

### Neutrophils

About a dozen studies have addressed the effects of serotonin on neutrophils, but the results are rather controversial, including diametrically contradictory findings. To date, the existence or non-existence of serotonergic components in neutrophils has not been confirmed. Some groups propose direct serotonin effects on neutrophils while others have attributed serotonin effects on neutrophils to the release of messengers from endothelial cells, namely eicosanoids, or to direct extracellular effects of serotonin in oxidative burst (63–65).

#### Serotonin Attenuates Oxidative Burst

Different groups examined the production of reactive oxygen species (ROS) in neutrophils upon serotonergic stimulation. Simpson et al. found in 1991 that serotonin did not induce significant superoxide production in human neutrophils that were isolated with a Ficoll–Hypaque gradient (66). They noted, however, that, in contrast, serotonin inhibited neutrophil superoxide production upon stimulation with the chemotactic peptide fMLP at high concentrations (10 and 100 µM). In accordance, Jancinová et al. found in 2001 and 2003 that platelet-derived serotonin decreased ROS production by stimulated polymorphonuclear cells (67, 68). In 2007, Cíz et al. described that serotonin inhibited the oxidative burst of human phagocytes in whole blood in a 5-HT2 receptor-dependent manner (69). The authors proposed that this inhibitory serotonin effect may be mediated in part by the inhibition of the neutrophil lysosomal enzyme myeloperoxidase (as shown by myeloperoxidase activity inhibition in a promyelocytic cell line). In addition, Cíz’s group and others showed that serotonin can directly scavenge ROS at concentrations above 10 µM (63, 69, 70). Schuff-Werner et al. described in 1995 that serotonin acted as a scavenger of ROS and was oxidized to a homodimer during the respiratory burst in mononuclear and polymorphonuclear cells (63). In contrast to descriptions of serotonin effects on neutrophils, Pracharova et al. reported in 2010 that serotonin inhibited the oxidative burst in total leukocyte preparations from human blood, but not in isolated neutrophils (71).

Taken together, these data indicate that serotonin may decrease the production of ROS by stimulated neutrophils. Still, the study by Pracharova et al. discusses the possibility that other cell types in non-pure polymorphonuclear leukocyte preparations may have mediated these effects. In addition, serotonin’s ROS-scavenging properties may primarily lie in a direct interaction between serotonin and ROS rather than cell-mediated effects. In conclusion, the question whether ROS production in neutrophils is modulated by serotonin has not been answered conclusively.

#### Serotonin Influences Neutrophil Recruitment

In 1996, Simonenkov et al. examined the chemotactic properties of non-specifically isolated human neutrophils in medium-filled chambers and found that addition of serotonin increased their movement velocity (72). In contrast, Bondesson et al. described in 1993 that while serotonin inhibited migration of Ficoll-gradient-separated mononuclear leukocytes (predominantly lymphocytes and monocytes), it had no effect on polymorphonuclear cells (mostly neutrophils) (73).

Other conflicting reports describe the interaction between leukocytes and (inflamed) endothelium upon serotonergic intervention. Doukas et al. found in 1987 and 1989 that treatment of cultured calf aortic endothelial cells with serotonin increased intercellular integrity and decreased the motility of polymorphonuclear cells (predominantly neutrophils) (64, 65). In contrast, Kubes et al. showed in 1996 that perivascular mast cells secrete serotonin to induce the early, leukocyte-independent phase of edema formation (28). The recruitment of leukocytes (mostly neutrophils in the early phases) did not depend on mast cell-derived serotonin. When superperfusing mesentery venules with serotonin, Kubes and coworkers found that vascular permeability increased but observed neither rolling nor adhesion of leukocytes. Then again, in 2007, Walther et al. presented evidence in the other direction: early leukocyte adhesion (within 60 min, i.e., at least in a significant proportion neutrophils) after injection of bacterial endotoxin depended on the activation of serotonin receptors as shown by pharmacological blockade (30). Our group showed in 2013 that platelet serotonin enhances neutrophil recruitment to sites of inflammation and there was less neutrophil recruitment in the absence of serotonin (32).

#### Serotonin May Affect the Phagocytic Activity of Neutrophils

In a study by Nannmark et al. from 1992, direct treatment of polymorphonuclear leukocytes with serotonin suppressed tumor cell- and zymosan-induced phagocytosis in a chemiluminescence assay suggesting a possible negative role for serotonin in tumor cell destruction (74). These findings may explain the observation by Skolnik et al. (1985) that thrombocytopenia and 5-HT2 receptor antagonism with ketanserin both decreased the liver invasion by injected tumor cells (75). In contrast, as early as in 1961, Northover saw that serotonin stimulated polymorphonuclear cell phagocytosis of staphylococci (76).

When Schuff-Werner and Splettstoesser examined general biological functions of human polymorphonuclear cells after treatment with serotonin in 1999, they found complex dose-dependent responses upon challenge with opsonized *Staphylococcus aureus* (77): at physiological serotonin concentrations (1–10 µM), the antibacterial defense improved significantly (may be due to reduced autooxidation), whereas higher concentrations (1–10 mM) counteracted an efficient bacterial killing.

In conclusion, several studies suggest specific responses of neutrophils to serotonergic stimulation—and hence the presence of at least one serotonin receptor. To date, however, this has not been examined to the best of our knowledge. According to the presented studies, the main effect of serotonin on neutrophils may be the improvement of autooxidation and the suppression of oxidative burst, but neutrophil recruitment may also be influenced.

### Eosinophils

Eosinophils express 5-HT1A, 5-HT1B, 5-HT1E, 5-HT2A, 5-HT2B, and 5-HT6 receptors with 5-HT2A being the most predominantly expressed (78). *Via* the 5-HT2A receptor eosinophil recruitment, airway inflammation, airway hyperresponsiveness, and remodeling is mediated in allergic asthma (79–81). In 2013, Kang et al. showed that serotonin induces eosinophil trafficking and recruitment *via* activation of ROCK, MAPK, PI3K, and the PKC-calmodulin pathway (78). Selective activation of the 5-HT2A receptor with (R)-DOI prevents eosinophil recruitment in an asthma model (82).

### Basophils/Mast Cells

Mast cells in rodents are described to be an important source of serotonin, while in humans, serotonin is normally absent in mast cells (or at least found in very low concentrations). Serotonin is only found in mast cells in nameable concentrations in humans in discrete pathologies such as in the stroma of carcinoid tumors or in mastocytosis (16, 83, 84). Rodent and human mast cells express the enzyme Tph1, so that they are capable to produce serotonin and release it to form early edema in inflammation (28, 85). One study of Crivellato and colleagues in 1991 even suggests that mast cells can release serotonin in part under neural control from nerve fibers containing substance P, calcitonin gene related peptide, vasoactive intestinal polypeptide, and somatostatin in the rat mesentery with a close anatomical relationship (17). In 2006, a study of Kushnir-Sukhov and colleagues showed that mast cell adhesion and migration in human and rodent mast cells is induced by serotonin *via* the 5-HT1A receptor (86).

### Lymphocytes

Lymphocytes take up serotonin *via* SERT (36, 87). Interestingly, serotonin uptake *via* SERT drove apoptosis of Burkitt lymphoma cells, which could be reversed by SSRIs, in a study by Serafeim et al. in 2002 (88).

In 2000, Stefulj et al. measured mRNA levels in spleen, thymus, and peripheral blood lymphocytes of the rat and found significant levels of 5-HT1B, 5-HT1F, 5-HT2A, 5-HT2B, 5-HT6, and 5-HT7 receptor mRNA (89). 5-HT3 receptor mRNA was only detected after stimulation with mitogens.

### T-Cells

T-cells express numerous 5-HT receptors as well as all other serotonergic components (TPH1, SERT, and MAO) (48, 87, 89, 90).

In 1991, Askenase and colleagues described that serotonin released from mast cells and activating 5-HT2 receptors on recruited T-cells initiates the delayed-type hypersensitivity (91). In 1997, the same group showed that local secretion of serotonin from platelets initiates T-cell-dependent contact sensitivity mediated by IgE antibodys (92).

T cell proliferation involves 5-HT2 receptors (93). The stimulation of the 5-HT1A receptor on T-cells was shown to increase cell survival and S-phase transition by increased translocation of NFκB to the nucleus (94). In 2007, Léon-Ponte et al. showed that naive T-cells express predominantly the 5-HT7 receptor and T-cell activation was hereby enhanced through serotonin stimulation (95). Activation of the 5-HT2A receptor subtype, which is expressed in high levels in activated T-cells, with (R)-DOI represses T helper cell 2 (Th2) gene expression in allergic asthma (82).

### B-Cells

In 1995, Iken and colleagues described that mitogen-stimulated B-cell proliferation was dependent on serotonin stimulation *via* the 5-HT1A receptor (24). Rinaldi et al. identified, in 2010, the expression of 5-HT3A in normal and neoplastic B-cells (96). In 2005, Meredith and colleagues showed that upon activation, B-cells exhibit a significant increase in SERT expression (97). As described above, serotonin uptake *via* SERT drives apoptosis in Burkitt lymphoma cells and could be reversed by selective serotonin reuptake inhibitors (88). Hernandez et al. showed accordingly that treatment with SSRIs increases the number of circulating B-cells (98). These findings show that serotonin influences the adaptive immune response.

### NK-Cells

As described above, serotonin suppressed interactions between monocytes and NK cells, leading to an increase of NK cell functions that are normally inhibited by monocytes, such as cytotoxicity and IFN-γ production in studies by Hellstrand and colleagues in the early 1990s (42, 46, 47). In 2008, Evans et al. showed that SSRIs enhanced the cytosolic functions of natural killer cells *in vitro* (99). Furthermore, 2 years later, Hernandez and colleagues showed that the long-term treatment with SSRIs enhanced the NK cell proliferation (98). The signaling mechanisms remain unclear.

## Serotonin Effects on Vascular Smooth Muscle Cells and Endothelial Cells

### Vascular Smooth Muscle Cells

In 1995, Ullmer et al. presented RT-PCR screening experiments with rat, porcine, human arterial, and venous vascular cells (100). The group found that vascular smooth muscle cells expressed 5-HT1D, 5-HT2A, 5-HT7, and in some experiments 5-HT2B receptor mRNA.

Watts et al. found in 2009 that serotonylation of α-actin is necessary for contraction in smooth muscle cells (101). Ito et al. described in 2000 that serotonin increased the synthesis and release of IL-6 from vascular smooth muscle cells *via* the 5-HT2A receptor (25). Yu et al. found in 2008, that activation of 5-HT2A receptors in primary aortic smooth muscle cells provided a very potent inhibition of TNF-alpha-mediated inflammation (26). The TNF-α-induced expression of intercellular adhesion molecule (ICAM)-1, vascular cell adhesion molecule-1, IL-6, nitric oxide (NO), nuclear factor-κB was inhibited by the 5-HT2A receptor agonist (*R*)-1-(2,5- dimethoxy-4-iodophenyl)-2-aminopropane [(*R*)-DOI] with an IC50 of 10–20 pM. This suggests a superpotent inhibitory serotonin effect and may constitute a continuous immunosuppressive mechanism at resting plasma serotonin concentrations.

### Endothelial Cells

Endothelial cells express 5-HT1B (in rodent) and accordingly 5-HT1Dβ (in other species), 5-HT2A, 5-HT2B, 5-HT2C, and 5-HT4 receptor mRNA (100). In 2001 and 2006, Eddahibi et al. presented evidence that pulmonary endothelial cells express SERT and TPH1 at increased levels in patients with idiopathic pulmonary artery hypertension (37, 102). Although at least 10-fold lower than in the intestine, the authors found significant expression of TPH1 also in the lung of healthy subjects.

The group around Shepro presented several studies in the 1980s to show that serotonin increased intercellular endothelial integrity (64, 65, 103, 104). The conclusion from these studies was that serotonin decreased polymorphonuclear leukocyte motility, diapedesis, and albumin permeability by decreasing eicosanoid release in *in vitro* experiments with calf aortic endothelial cells. In contrast to these findings, Kubes et al. measured increasing leakage of labeled albumin in living rats after serotonin-release from mast cells and exogenous serotonin administration in 1996 (28, 105). These studies supported the theory that serotonin is a key inductor of early edema formation.

Katz et al. found in 1994 that serotonin-stimulated endothelial cells secreted an unknown endothelial cell-derived T cell chemoattractant and growth factor (106). This indicates that serotonin might influence leukocyte recruitment by interacting with endothelial cells. Marconi et al. found in 2003 that blocking of 5-HT2 receptors with naftidrofuryl inhibited the TNF-α-triggered expression of ICAM-1 expression and stress fiber formation in human umbilical vein endothelial cells (*via* NO release) (107). Our group found in 2014 that E-selectin expression on endothelium was upregulated upon higher serotonin levels in plasma (33). Although direct serotonin effects cannot be extrapolated from these results, it is possible that serotonin is involved in the regulation of endothelial adhesion receptor expression. Stimulation of 5-HT2B receptor induced endothelial nitric oxide synthase expression in human umbilical vein endothelial cells in a study by Asada et al. from 2009 suggesting that this may play an important role in tumor angiogenesis (108).

In conclusion, serotonin may regulate leukocyte recruitment and vascular integrity in inflamed vessels by modulating endothelial cells.

## Conclusion

This review shows the complex influences of serotonin on immune cells. So far, we don’t know and understand all underlying mechanisms, but it gets clearer, that the neurotransmitter and the peripheral hormone serotonin plays an important role in immunity and in inflammatory and immunomodulatory diseases. No matter if the serotonin derives from platelets, mast cells, T-cells, or even from neurons. For example, it influences diseases like gut inflammation (109), allergic asthma (110), rheumatoid arthritis (111), and neuroinflammation such as ALS (60) and autism (112). In the recent years, we could observe the anti-inflammatory effects of antidepressant (influencing serotonin levels in the body) in clinical practice that are, for example, mediated *via* macrophage modulation (113). Serotonin seems to be a promising new target when it comes to modulating immune responses.

## Author Contributions

NH and DD wrote the manuscript. CB provided helpful advice and critical reading of the paper.

## Conflict of Interest Statement

The authors declare that the research was conducted in the absence of any commercial or financial relationships that could be construed as a potential conflict of interest.
